# A rare presentation of linea alba hernia involving fibrolipoma of the hepatic round ligament: a case report and literature review

**DOI:** 10.1186/s40792-023-01676-x

**Published:** 2023-05-29

**Authors:** Takahiko Omameuda, Mikio Shiozawa, Yuzo Miyahara, Hiroyuki Kitabayashi, Masaru Koizumi, Satoru Kondo, Shigeo Kawai, Masaaki Kodama

**Affiliations:** 1 Department of Surgery, Tochigi Medical Center Shimotsuga, Ohiramachi Kawazure 420-1, Tochigi, Tochigi 329-4498 Japan; 2 Department of Pathology, Tochigi Medical Center Shimotsuga, Ohiramachi Kawazure 420-1, Tochigi, Tochigi 329-4498 Japan

**Keywords:** Aged, Adipose tissue, Abdominal wall, Abdominal pain, Linea alba hernia, Fibrolipoma, Hepatic round ligament, Cell proliferation, Tomography

## Abstract

**Background:**

Linea alba hernias are relatively rare types of hernias. They manifest as small protrusions situated in the linea alba between the umbilicus and xiphoid cartilage. Usually, hernia contents comprise the pre-peritoneal fat, omentum, and gastrointestinal tract. However, very few cases of linea alba hernias involving the hepatic round ligament have been reported, to date.

**Case presentation:**

An 80-year-old woman presented with upper abdominal pain and a 1-week history of a mass in the upper midline. Abdominal computed tomography revealed adipose tissue protruding from the abdominal wall contiguous with the hepatic round ligament, suggesting a linea alba hernia. During surgery, the hernial sac content was found to be a mass, which was resected. A linea alba hernia defect measuring 20 mm was repaired using a mesh. Histopathological findings revealed that the mass included mature adipocyte proliferation with broad fibrous septa, which was diagnosed as fibrolipoma of the hepatic round ligament.

**Conclusions:**

We report the first case of a linea alba hernia involving fibrolipoma of the hepatic round ligament worldwide and describe the clinical features, diagnosis, and surgical procedure with a literature review.

## Background

Linea alba hernias are relatively rare hernias with predominance in older adults, particularly female patients. Linea alba hernias have been reported to represent only 3.6% of all hernias in Europe and the United States, with lower incidence in Japan [1]. Furthermore, the hernia contents usually include the pre-peritoneal fat, omentum, and gastrointestinal tract, with very few cases of linea alba hernias involving the hepatic round ligament reported. In this report, we describe a case in which the hernia content included fibrolipoma of the hepatic round ligament.

## Case presentation

An 80-year-old woman with no history of abdominal surgery visited our hospital because of persistent upper abdominal pain and a 1-week history of a swelling. The patient was 145.7 cm tall and weighed 55.8 kg, resulting in a body mass index (BMI) of 26.3 kg/m^2^. She had one previous pregnancy and childbirth.

**Fig. 1:**
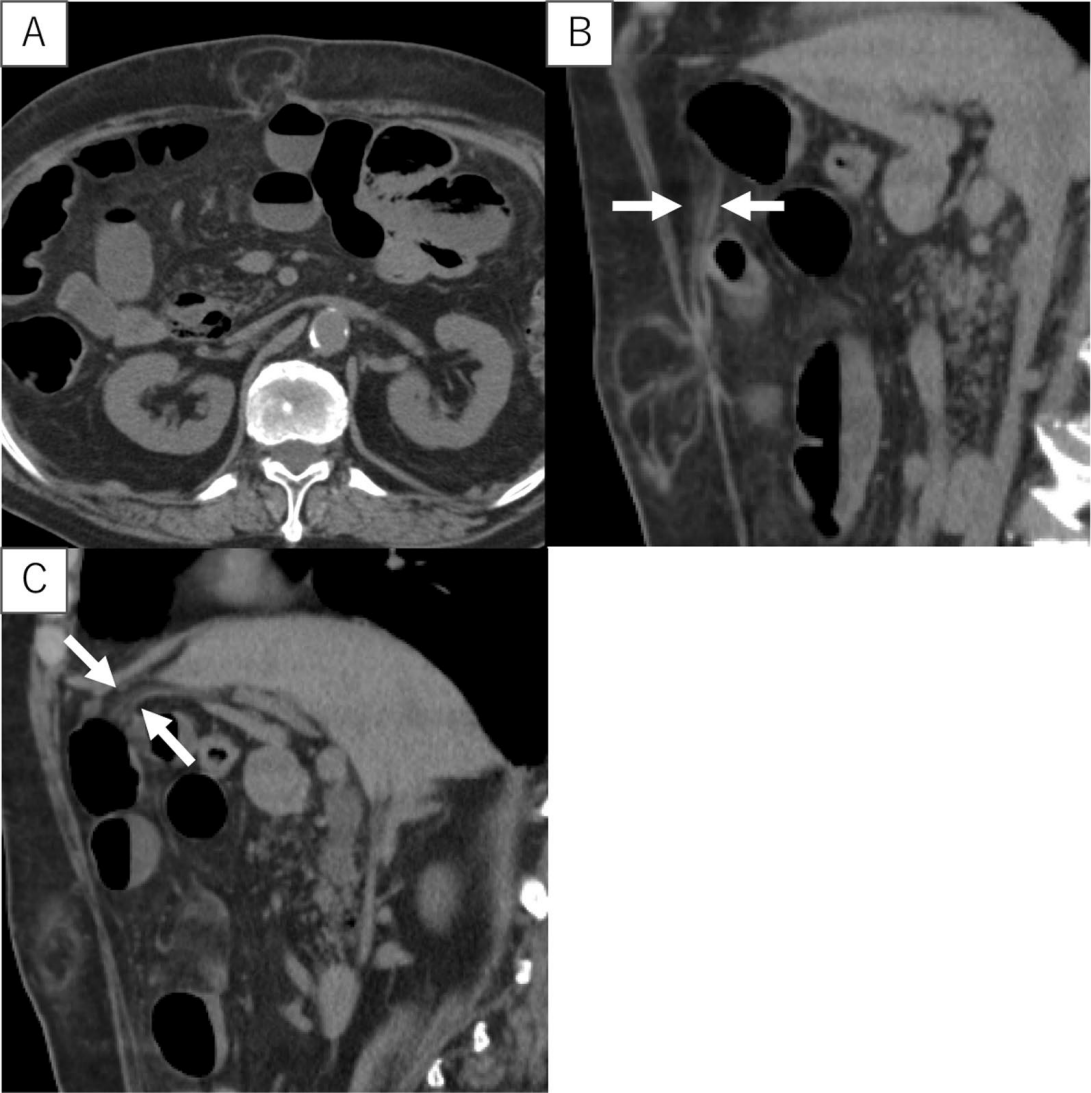
Computed tomography scan. **A** Adipose tissue herniated from the linea alba. **B**, **C** Adipose tissue contiguous with the hepatic round ligament (white arrow)

Physical examination revealed an elastic soft bulging mass, 8 cm in diameter, in the midline of the upper abdomen, with a zone of defect along the linea alba, without signs of peritoneal irritation. At the time of the patient’s initial ambulance visit, manual reduction of the intestinal tract was successfully achieved. However, repositioning of some hernia contents was deemed impracticable. Blood tests revealed slightly elevated white blood cell count and decreased renal function due to dehydration. Abdominal computed tomography (CT) showed adipose tissue contiguous with the hepatic round ligament with herniation through the linea alba (Fig. 1A–C).

**Fig. 2:**
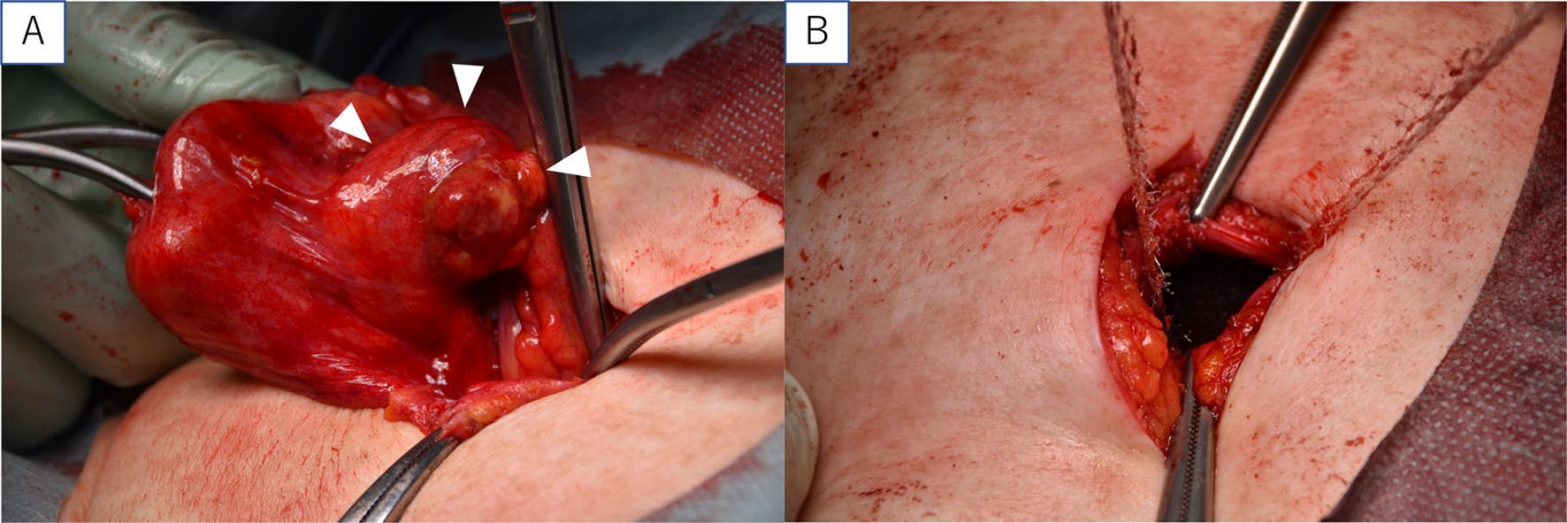
Surgical observations. **A** White arrowheads indicate a mass contiguous with the hepatic round ligament and a thickened pre-peritoneal fat. **B** Mesh is inserted into the abdominal cavity

Since manual reduction of the mass led to symptom improvement, elective surgery was subsequently performed. Following an incision in the skin just above the hernial orifice (20 mm), a mass contiguous with the hepatic round ligament and thickened pre-peritoneal fat surrounding the mass was observed (Fig. 2A). Resection of the mass and hepatic round ligament was performed. Ventralex™ ST Hernia Patch (BD, RI, USA) was then inserted into the abdominal cavity (Fig. 2B).

**Fig. 3:**
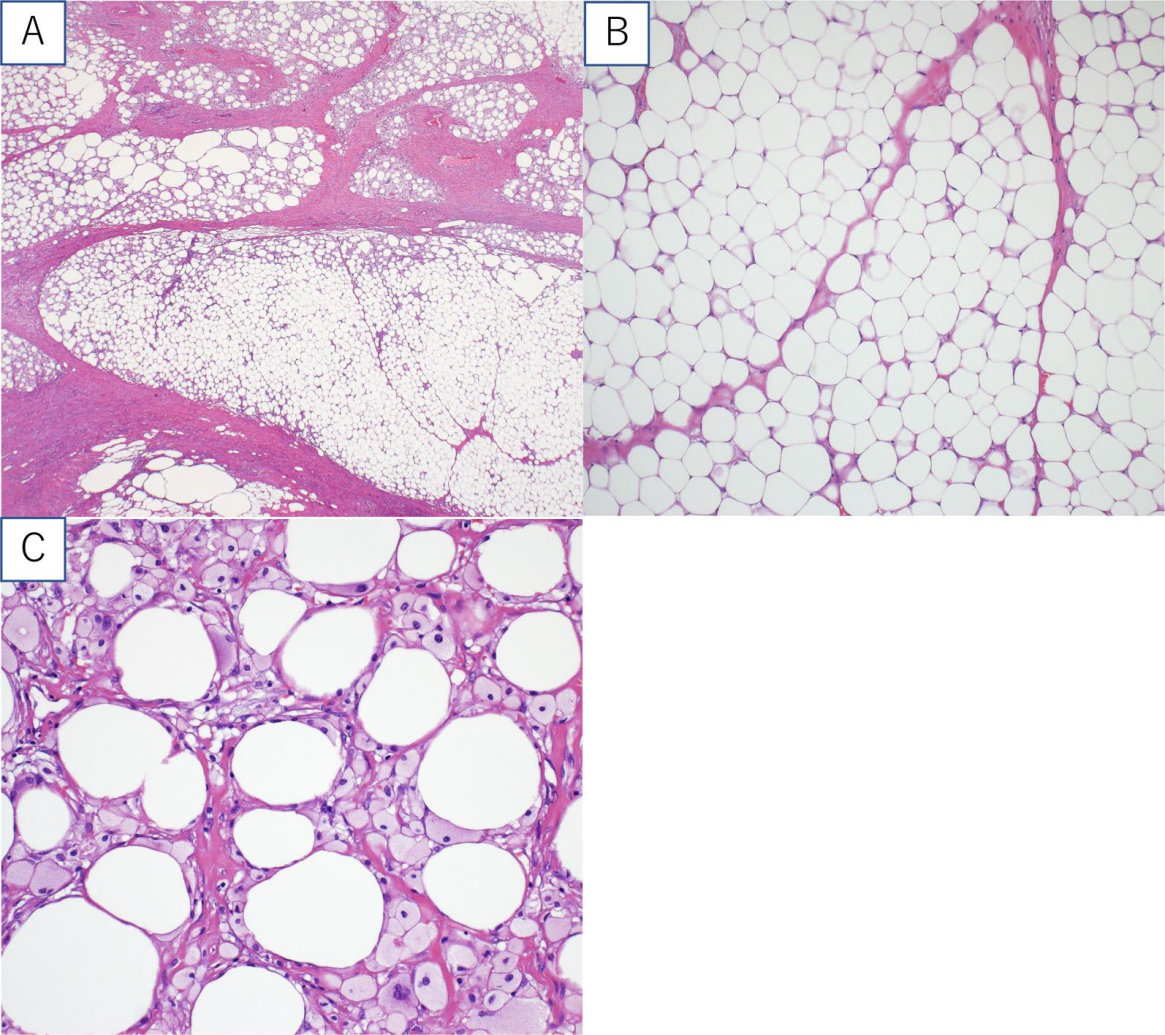
Pathological findings (hematoxylin and eosin stanning). **A** Proliferation of mature adipocytes and intersecting broad fibrous bands, **B** cytological atypia is absent. **C** Necrosis of adipose tissue and invasion of lipophage

Pathological findings showed that the tumor consisted of mature adipocyte proliferation, with intersecting broad fibrous bands (Fig. 3A). Cytological atypia was absent (Fig. 3B). The yellow-white areas of the tumor margins showed necrosis of adipose tissue and lipophage invasion (Fig. 3C), suggesting necrosis due to incarceration of the tumor. Hence, the patient was diagnosed with fibrolipoma of the hepatic round ligament, characterized by the presence of numerous fibrous elements in a lipoma. The patient was discharged on postoperative day 5 and has been without recurrence for 2 years.

## Discussion

Linea alba hernias are rare in Japan. Less than 100 cases have been reported since Kumagaya et al. first reported it in 1923 [1]. The etiology of linea alba hernias is likely multifactorial. It may include (i) a congenitally weak portion of the linea alba; (ii) increased intra-abdominal pressure due to obesity, pregnancy, or ascites; (iii) pre-peritoneal fat tissue causing a defect in the linea alba; (iv) anatomical variants of the abdominal wall musculature; and (v) trauma [2, 3].

The patient exhibited mild obesity with a BMI of 26.3 kg/m^2^. She had a history of a single pregnancy and delivery, without a history of trauma. Intraoperatively, the hernia’s development was considered to be primarily due to the abnormal proliferation of pre-peritoneal adipose tissue and resultant defect in the linea alba, rather than obesity or pregnancy history. Linea alba hernias contain the pre-peritoneal fat, omentum, and small intestine in 30.6% of cases, colon in 11.8%, stomach in 7.1%, and falciform ligament in 2.4% [4]. Multiple hernial orifices have been reported in approximately 20% of patients with linea alba hernias [5]. To our best knowledge, among linea alba hernias, there are few cases of hernias involving the hepatic round ligament.

Linea alba hernias in the upper abdominal region are sometimes classified as epigastric hernias. We searched PubMed using the terms “linea alba hernia”, “epigastric hernia”, “hepatic round ligament, and “fibrolipoma”, and identified no publications with those terms. To our best knowledge, except for our case, we found no cases of fibrolipoma of the hepatic round ligament with herniated content in linea alba hernias.

In Japan, there have been nine cases [4, 6–12] in which the hernia contents included the hepatic round ligament, including our case (Table 1); the average age was 68.4 years and eight patients were female. The average size of the hernial orifice was 19.4 mm. Four patients underwent open repair, and five underwent laparoscopic repair. Repaired with mesh in four cases and without mesh in five cases, hernia was not reported to recur in any of the cases.

**Table 1:** Cases of linea alba hernia caused by hepatic round ligament

Case	Reporter	Reported year	Sex	Age	Hernia content	Hernia orifice (mm)	Incarceration	Other hernia comorbidities	Operation	Recurrence
1	Yoshida et al.	1997	F	77	Hepatic round ligament	20	No	N/A	Open laparotomy with direct sutures	Not
2	Miyaso et al.	2010	F	78	Hepatic round ligament	30	Incarcerated	N/A	Open laparotomy with direct sutures	Not
3	Matsui et al.	2017	F	53	Hepatic round ligament	10	N/A	No	Laparoscopic repair with direct sutures	Not
4	Nonoyama et al.	2017	F	70	Hepatic round ligament	25	Incarcerated	N/A	Open laparotomy with direct sutures	Not
5	Nagao et al.	2020	F	57	Hepatic round ligament	10	Incarcerated	N/A	Open laparotomy with direct sutures	Not
6	Saitou et al.	2020	F	55	Hepatic round ligament	20	Incarcerated	N/A	Laparoscopic repair with mesh	Not
7	Nishi et al.	2020	F	76	Hepatic round ligament	10	Incarcerated	No	Laparoscopic repair with mesh	Not
8	Takii et al.	2021	M	70	Hepatic round ligament	30	Incarcerated	N/A	Laparoscopic repair with mesh	Not
9	Omameuda et al.	2022	F	80	Fibrolipoma of hepatic round ligament	20	No	Unknown	Open laparotomy with mesh	Not

*M* male, *F* female, *N/A* not available

Laparoscopic surgery necessitated the insertion of at least two ports, with one serving as a camera port. Because the patient was not in the postoperative phase and CT imaging suggested the unlikelihood of presence of multiple hernia sacs, intra-abdominal observation using a laparoscope was deemed superfluous. Single-port laparoscopic surgery in linea alba hernia has become reported in recent years [10]. There are some limitations to single-port laparoscopic surgery, such as reduced instrument mobility, it has been shown that single-port laparoscopic surgery group had a lower rate of postoperative complications, higher rate of intraoperative complications rather than conventional laparoscopic surgery group in colorectal surgery [13]. Single-port laparoscopy was deemed a viable alternative, if the institution possessed proficient practitioners in the technique, specifically for linea alba hernias. However, the author’s lack of familiarity with single-port laparoscopy for linea alba hernias precluded its use in this instance. Consequently, open surgical repair was selected because the experience gained from treating abdominal incisional hernias and similar conditions can be applied.

Regarding the use of mesh in abdominal wall hernias, there have been reports of significantly fewer recurrences with the use of mesh than with simple suture closure in cases of small hernia orifices of less than 20 mm [14]. Based on this evidence, we elected to incorporate mesh during the closure procedure. A meta-analysis between the laparoscopic and open groups for abdominal wall hernias found no significant difference in recurrence between the two groups [15].

Among the primary tumors of the hepatic round ligament, there are several reported cases of tumors such as cystic lymphangioma [16], fibroma [17, 18], hemangioendothelioma [19], hepatocellular carcinoma [20], leiomyoma [21], leiomyosarcoma [22], and liposarcoma [23]. Since tumor is often malignant, its resection, including the round hepatic ligament, is preferred. In this case, considering the possibility of malignancy, the tumor, including the round hepatic ligament, was resected. The specimen was pathologically diagnosed as a fibrolipoma of the hepatic round ligament.

If a tumor of the hepatic round ligament is noted intraoperatively, resection of the hepatic round ligament is considered necessary, and a pathological diagnosis of the specimen is required.

## Conclusion

Herein, we report the first case of linea alba hernia with fibrolipoma of the hepatic round ligament as the hernia content that was repaired using a mesh. Since tumors of the hepatic round ligament may be malignant, resection involving the hepatic round ligament was considered necessary, and the specimen was pathologically diagnosed.

## Data Availability

Not applicable.

## Abbreviation

CT: Computed tomography

## References

[1] Sakamoto T, Kawamoto J, Nishida T (2018). A case of linea alba hernia occurring in a nonobese type body. Chiba Med J.

[2] Ponten JE, Somers KY, Nienhuijs SW (2012). Pathogenesis of the epigastric hernia. Hernia.

[3] Arai T, Matsushita A, Kubo M, Kumaki T, Kasuga Y (2005). A case of traumatic hernia in the linea alba in an aged patient. Nihon Rinsho Geka Gakkai Zasshi (J Jpn Surg Assoc).

[4] Miyaso H, Nishie M, Iwagaki H (2005). A case of linea alba hernia with impaction of the falciform ligament of liver. J Okayama.

[5] Igari K, Ochiai T, Tokairin Y (2009). A case of multiple linea alba hernia. Nihon Rinsho Geka Gakkai Zasshi (J Jpn Surg Assoc).

[6] Yosida S, Tomita T, Kogure H (1997). A case of epigastric hernia. Dokkyo J Med Sci.

[7] Matsui R, Kitamura H, Yamamoto D (2017). A case of linea alba hernia with prolapsed hepatic round ligament; that had difficulty in preoperative diagnosis. Geka.

[8] Nonoyama K, Hayakawa T, Takashima N, Yamamoto M, Kitagami H, Tanaka M (2017). Linea alba hernia repair by laparoscopic transabdominal preperitoneal approach. Nihon Rinsho Geka Gakkai Zasshi (J Jpn Surg Assoc).

[9] Nagao M, Mori S, Maeda N, Sano T, Okada S (2020). A case of emergency surgery for a linea alba hernia with prolapsed hepatic round ligament. Nihon Rinsho Geka Gakkai Zasshi (J Jpn Surg Assoc).

[10] Saito A, Haruta H, Kumagai Y, Kono Y, Lefor AK, Sata N (2019). Single incision laparoscopic surgery repair of a linea alba hernia. Jichi Med Univ J.

[11] Nishi Y, Ito Y, Seo Y (2022). Laparoscopic repair of linea alba hernia associated with hepatic round ligament incarceration—a case report. Nihon Rinsho Geka Gakkai Zasshi (J Jpn Surg Assoc).

[12] Takii M, Takemura M, Gyobu K (2021). Two cases of linea alba hernia treated by laparoscopic transabdominal preperitoneal technique. J Jpn Soc Endoscopi Surg (JSES).

[13] Yuan Y, Jian J, Jing H, Yan R (2021). Single-incision vs. conventional laparoscopic surgery for colorectal cancer: an update of a systematic review and meta-analysis. Front Surg.

[14] Christoffersen MW, Helgstrand F, Rosenberg J, Kehlet H, Bisgaard T (2013). Lower reoperation rate for recurrence after mesh versus sutured elective repair in small umbilical and epigastric hernias. A nationwide register study. World J Surg.

[15] Zhang Y, Zhou H, Chai Y, Cao C, Jin K, Hu Z (2014). Laparoscopic versus open incisional and ventral hernia repair: a systematic review and meta-analysis. World J Surg.

[16] Kohno M, Takahashi S, Oshikiri T (2008). Cytic lymphangioma of round ligament of the liver in a child. Nihon Shoni Geka Gakkai Zasshi (J Jpn Soc Pediatr Surg).

[17] Beyer L, Delpero JR, Chetaille B, Sarran A, Perrot D, Moureau-Zabotto L (2012). Solitary fibrous tumor in the round ligament of the liver: a fortunate intraoperative discovery. Case Rep Oncol.

[18] und Torney MV, Brunner P, von Holzen U, Hohmann J, Kettelhack C (2014). A large fibroma of the round ligament of the liver. Surgery.

[19] Sotiropoulos GC, Moskalenko V, Lang H. Clinical challenges and images in GI. Image 2. Perivascular epithelioid cell (PEC) tumor of the ligamentum teres. Gastroenterology. 2009;136:2065, 2416. 10.1053/j.gastro.2009.01.045. 10.1053/j.gastro.2009.01.045 19427317

[20] Kim SY, Lim JH (1985). Extension of hepatoma to the rectus abdominis muscle via ligamentum teres hepatis. Gastrointest Radiol.

[21] Matito-Díaz MJ, Blanco-Fernández G, Fernández-Pérez J, López-Guerra D (2015). Leiomyoma of the round ligament of the liver: report of one case. Rev Esp Enferm Dig.

[22] Yamagiwa H, Onishi C, Kobayashi M (1967). Leiomyosarcoma of the suspensory ligamentum of the liver. Mie Med J.

[23] Tsuchiya Y, Takeda K, Nikaido T, Yamada T, Akimaru K, Uchida E (2013). A case of liposarcoma developing in the ligamentum teres hepatis concomitant with rectosigmoid cancer. J Nippon Med Sch.

